# Is There Any Practical Application of Meta-Analytical Results in Strength Training?

**DOI:** 10.3389/fphys.2017.00001

**Published:** 2017-01-19

**Authors:** Paulo Gentil, Antonio Arruda, Daniel Souza, Jurgen Giessing, Antonio Paoli, James Fisher, James Steele

**Affiliations:** ^1^Faculdade de Educação Física e Dança, Universidade Federal de GoiásGoiania, Brazil; ^2^Laboratório de Pesquisas em Desempenho Humano, Universidade de Pernambuco-Campus PetrolinaPetrolina, Brazil; ^3^Departamento de Educação Física, Universidade Federal do Rio Grande do NorteNatal, Brazil; ^4^Institute of Sport Science, University of Koblenz-LandauLandau, Germany; ^5^Department of Biomedical Sciences, University of PadovaPadova, Italy; ^6^Sport Science Laboratory, Centre for Health, Exercise and Sport Science, Southampton Solent UniversitySouthampton, UK

**Keywords:** strength training, exercise prescription, strength and conditioning, resistance exercise, muscle hypertrophy, muscle strength

## Introduction

Designing resistance training (RT) programs is a complex task that involves the manipulation of numerous variables that interact with each other, influencing the program outcomes (Tan, 1999; Paoli, 2012). The attempt to clearly define the combination of variables which would bring optimal adaptations for different outcomes is undermined by the large number of studies involving RT, the conflicting findings reported by many of them and the lack of methodological clarity and consistency in previous studies' protocols. As such, meta-analyses emerge as an attractive approach since they allow the combination of multiple studies in an attempt to estimate the effect size of a single variable, surpassing possible inadequacies of statistical power within individual studies. With this aggregation of information, a more robust estimation of the effects is possible.

However, Field (2015) has noted a pertinent philosophical objection to these types of analyses that might apply to RT studies; in essence we have a replication crisis. Researchers often attempt to perform replications of the findings from earlier studies, yet frequently they do not adequately replicate the conditions of the original study. For example, one study may examine the effects of low or high set volume whilst participants train at a frequency of twice a week using repetition ranges of 8–12 and perform sets to momentary failure. Another may examine the effects of low or high set volume whilst participants train at a frequency of five times a week using 10 repetitions per set and not having participants perform sets to momentary failure. Though the two studies might appear to be examining whether low or high set volumes produce greater adaptations, they are in fact examining these within the context of different manipulations of other RT variables. There is likely a reason for this lack of proper replication, as was noted by Richard Feynman^1^. Indeed, we would argue that the currently heterogeneous body of literature on the effects of the manipulation of different RT variables is evidence of this replication crisis being alive and well in our field.

In this current opinion article we explain specifically why it might be unwise to conduct meta-analyses with such heterogeneous RT studies noting the effects of different confounding RT variables, and also suggest that it might be irresponsible to make general estimates of RT effects and propose recommendations.

## Impact of resistance training variables

The first aspect to consider is the confusion borne from the definition and reporting of training “*intensity*.” RT studies have previously defined and thus controlled exercise “*intensity”* as a percentage of the load equivalent to the one repetition maximum (1RM). Nevertheless, previous studies reported that the number of repetitions performed at a given percentage of 1RM largely varies when different individuals are performing the same exercise and when the same individual performs different exercises (Hoeger et al., 1990). Hoeger et al. (1990) reported that, when using 80% of 1RM, resistance trained women were able to perform 22 repetitions in the leg press exercise and only 9 in the knee extension. Therefore, if a study prescribes 12 repetitions at 80% of 1RM for the leg press and another prescribes the same repetitions and load for the knee extension, they would be performing the exercise with a similar intensity of load but different intensity of effort. Additionally, Hoeger et al. (1990) reported the average number of repetitions performed with 80% of 1RM was 22 for trained and 12 for untrained women. As a result, the prescription of the same number of repetitions at the same relative load would result in different intensity of effort. For this reason, several authors have proposed that using 1RM percentages may be inadequate for controlling RT intensity (Fisher and Smith, 2012; Fisher et al., 2013; Steele, 2014) and suggested that controlling effort (i.e., exercising to the point of momentary failure) would be a better strategy for this purpose.

The need for controlling effort is further supported by a previous study reporting that training to failure results in greater gains in muscle strength and more positive changes in body composition than not training to failure (Giessing et al., 2016). Considering this, the use of percentages of 1RM in many of the previous studies might have resulted in unmatched intensities of effort, which likely influenced training outcomes. We acknowledge that this is a contentious issue, and other studies exist suggesting that, where volume matched, equivocal results can be obtained whether training to muscular failure or not (Fisher et al., 2016c). However, other authors have suggested that training to muscular failure is necessary for standardizing RT interventions (Dankel et al., 2016a). With this in mind, we believe that the inadequate control of intensity of effort is a problem in many previous studies, since there is often no clear definition of the set end point used in training which was performed to either volitional fatigue, or a self-determined repetition maximum or to momentary failure.

Supervision ratio might be another aspect to influence the results of RT. In previous studies by Gentil and Bottaro (2010), Mazzetti et al. (2000), and Coutts et al. (2004), untrained men, trained men and young athletes had higher gains in muscle strength and performance when training with a more favorable supervision ratio (e.g., fewer participants to trainers). It is important to note that in these studies all participants performed the same protocol, with the same exercises, number of sets, repetitions, etc. The differences in the results were due to the supervision ratio. It is likely that a more favorable supervision ratio resulted in participants exercising to a greater intensity of effort. More recent studies which have used a one to one (client: trainer) ratio have failed to identify any benefits from performing advanced RT methods (Fisher et al., 2014, 2016a,b) likely because all participants, irrespective of group, exercised to true momentary failure due to the supervision. It is our opinion that whilst, supervision ratio is not usually considered in RT meta-analyses (e.g., Rhea et al., 2003; Peterson et al., 2004; Wernbom et al., 2007; Krieger, 2010; Schoenfeld et al., 2015, 2016a,b) possibly since it is often unreported in RT studies; it is an important factor which needs future consideration in both empirical trials and review articles including meta-analyses.

Other aspects usually forgotten are movement velocity and the type of muscle action predominantly performed. Previous studies suggest that different muscle actions influence the acute responses to exercise. For example, when using the same load, it is possible to perform more repetitions when only eccentric actions are performed in comparison to combined concentric and eccentric, which, in turn, permits greater repetitions than concentric only muscle actions (Flanagan et al., 2014). Moreover, eccentric actions have been shown to induce a higher degree of muscle damage than concentric, even when using the same load and performing the same number of repetitions (Gibala et al., 1995). Consequently, two persons performing the same number of sets and repetitions for the same exercise, at the same relative load may have different physiological responses if using different movement velocities in the concentric or eccentric phase. In addition, previous studies reported differences in anabolic signaling (Burd et al., 2012), fitness (Bottaro et al., 2007) and gains in muscle size and strength (Nogueira et al., 2009) when the same exercise protocol was performed with different velocities. Again, this is often an overlooked variable which we believe is important when considering studies to include in a meta-analysis.

Another possible source of confusion might be considering only specific muscles (e.g., the supposed prime movers) when counting the number of sets performed. This aspect is especially relevant for upper body muscles (i.e., not considering the biceps brachii work during pulldowns), since previous research has shown that upper-body multi joint exercises produce equivalent gains in muscle size and strength as single joint exercises specifically targeted for the elbow flexors (Gentil et al., 2015, 2016). Therefore, one may not disregard the involvement of a muscle in the multi joint movement (i.e., the involvement of triceps during bench press, biceps during pulldowns, etc.) as it will lead to an inadequate estimation of training volume. Moreover, including studies that measured legs and arm muscles in the same analyses could be misleading, Considering that previous data did not present the same trend of greater adaptations with increasing sets per muscle group across both upper- and lower-body muscles (Ostrowski et al., 1997; Paulsen et al., 2003; Ronnestad et al., 2007; Bottaro et al., 2011) we believe that dissociation of these muscle groups, rather than a single recommendation, might be beneficial. With the above in mind we believe that when performing meta-analyses authors can inadvertently misrepresent research studies and might exclude essential details which serves to reduce the validity of their conclusions.

In addition to the above variables which are often left unreported in research publications and uncontrolled in meta-analyses we should also consider the variable of range of motion (ROM). In previous studies, participants whose training differed only in ROM of the exercise performed (e.g., all other variables were controlled) showed between group variation in muscle size, strength, and decreases in skinfold thickness in favor of a larger ROM (Massey et al., 2005; Pinto et al., 2012; Bloomquist et al., 2013; McMahon et al., 2014). Thus, we believe that if ROM is not reported and controlled this may have differed between interventions and depending on the outcome examined may impact any conclusions.

Finally, the tests used to measure outcomes seem to be of vital importance for estimating effect size. When analyzing strength two of the most popular tests are the one-repetition maximum (1RM) and isokinetic or isometric peak torque (PT) (Brown and Weir, 2001). Although the two methods are widely used and accepted by the scientific community, they are not equivalent, as the results obtained show large variations and can be even conflicting (Gentil et al., in press). For example, the effect size of an intervention can be large when measuring its results by 1RM, while the effect size of another intervention can be low when evaluating it by isometric or isokinetic dynamometry. Nevertheless, this may not be reflective of the intervention protocols, but rather of the tests performed. Thus, using studies that utilized different assessment methods in the same analysis may produce inaccurate and misleading results. The same is true for *in vitro* and *in vivo* methods of assessing change in hypertrophy as discussed previously (Steele and Fisher, 2014; Fisher et al., 2016d). Furthermore, the use of effect sizes in general has recently been challenged (Dankel et al., 2016b), and since meta-analyses calculate overall results from this value we urge caution in meta-analyses which provide conclusions contradictory to a body of research.

## Conclusions

These observations are only a few examples of the complexity involved with RT prescription, discussed to illustrate how isolated variables may influence RT outcomes. As such we question the possibility of making general estimates of RT adaptation without considering such complexity. The question is, if one single variable can influence the results, how can we control the interaction between them or how can we precisely estimate the impact of one of them when the others are not controlled?

A common criticism of meta-analyses is that they usually combine studies that have important methodological differences and, consequently, the summary effect can be largely influenced by these differences across studies (Field, 2015; Fuhr and Hellmich, 2015). Although there are many possible strategies to test for heterogeneity across included studies, and methods to account for such heterogeneity and confounding factors upon effect sizes (e.g., treating meta-analysis as a multi-level model), the power to test for these moderators depends partly on the number of studies available and the sample sizes used across these studies. For this reason, an effort for controlling confounding factors would be fruitless if the analyses involve a low number of heterogeneous studies with small sample sizes, as is the case in almost all RT meta-analyses. It is also important to note that a meta-analysis is only as good as the studies included. Thus an initial flaw in screening and selection can lead to inappropriate inclusion and/or analysis of inadequate studies, leading to the phenomenon: “garbage in garbage out” (Charlton, 1996).

Meta-analyses are highly publishable and have negligible cost and effort when compared to the acquisition of raw data. These might explain the increase in meta-analyses production and publication in many areas (Field, 2015; Fuhr and Hellmich, 2015), including RT. However, it is important to remember that for a meta-analysis to be valid, a large amount of data on homogeneous subgroups under homogenous conditions should accumulate for topics where there is strong consensus about which variables have theoretical importance, and this does not seem to be the case for RT. Therefore, we consider that a greater value can be obtained by designing and conducting studies of larger and homogenous samples that can adequately address the topics considered, or performing more exact replication studies instead of prematurely performing meta-analyses on differing RT variables or trying to estimate the effects of RT combining studies that involve an uncontrollable heterogeneity. If it is desirable to obtain an understanding of the conclusions that can be drawn from the body of existing literature, then it would be better to carefully review and interpret studies whilst considering the role of confounding variables and study designs.

## Author contributions

PG, AA, DS, JG, AP, JF, and JS: Conception, drafting the article, revising it critically, and final approval of the version to be published.

### Conflict of interest statement

The authors declare that the research was conducted in the absence of any commercial or financial relationships that could be construed as a potential conflict of interest.

## Footnotes

^1^“*…under certain circumstances, X, rats did something, A. She was curious as to whether, if she changed the circumstances to Y, they would still do, A. So her proposal was to do the experiment under circumstances Y and see if they still did A*. *I explained to her that it was necessary first to repeat in her laboratory the experiment of the other person—to do it under condition X to see if she could also get result A—and then change to Y and see if A changed. Then she would know that the real difference was the thing she thought she had under control*. *She was very delighted with this new idea, and went to her professor. And his reply was, no, you cannot do that, because the experiment has already been done and you would be wasting time. This was in about 1935 or so, and it seems to have been the general policy then to not try to repeat psychological experiments, but only to change the conditions and see what happens.”*(http://calteches.library.caltech.edu/51/2/CargoCult.htm)
